# Supplementary figures and images for: Altered co-stimulatory and inhibitory receptors on monocyte subsets in patients with visceral leishmaniasis
Source: PLoS Negl Trop Dis. 2024 Aug 19;18(8):e0012417. doi: 10.1371/journal.pntd.0012417 (PMC11373857; doi:10.1371/journal.pntd.0012417)

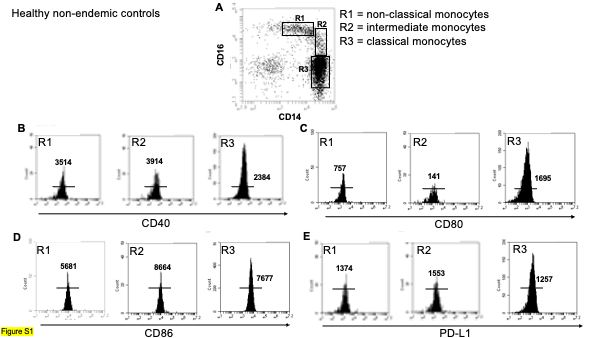

Supplement: S1 Fig — A. Identification of non-classical (R1), intermediate (R2) and classical (R3) monocytes based on the expression levels of CD14 and CD16. Histogram representing the expression level of CD40 (B), CD80 (C), CD86 (D) and PDL-1 (E) in non-classical (R1), intermediate (R2) and classical (R3) monocytes as measured by flow cytometry as described in Materials and Methods. One representative histogram is shown for each cell surface marker. (TIFF) [file pntd.0012417.s001.tiff]

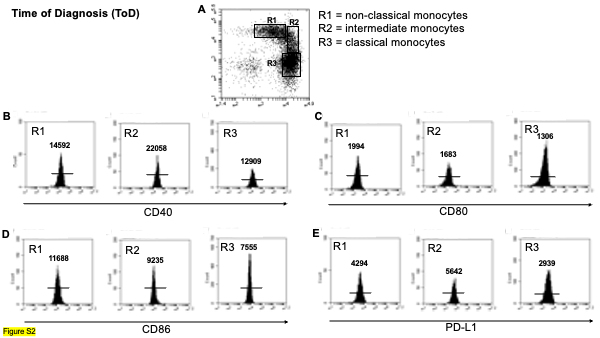

Supplement: S2 Fig — A. Identification of non-classical (R1), intermediate (R2) and classical (R3) monocytes based on the expression levels of CD14 and CD16. Histogram representing the expression level of CD40 (B), CD80 (C), CD86 (D) and PDL-1 (E) in non-classical (R1), intermediate (R2) and classical (R3) monocytes as measured by flow cytometry as described in Materials and Methods. One representative histogram is shown for each cell surface marker. (TIFF) [file pntd.0012417.s002.tiff]

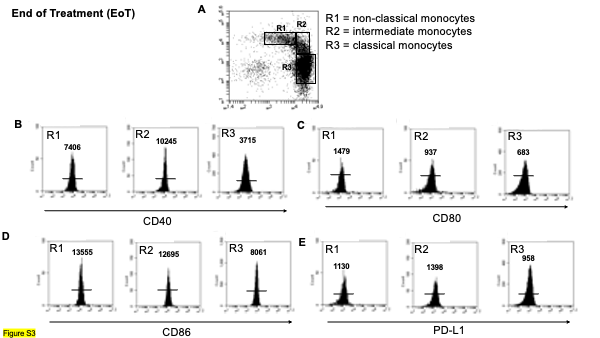

Supplement: S3 Fig — A. Identification of non-classical (R1), intermediate (R2) and classical (R3) monocytes based on the expression levels of CD14 and CD16. Histogram representing the expression level of CD40 (B), CD80 (C), CD86 (D) and PD-L1 (E) in non-classical (R1), intermediate (R2) and classical (R3) monocytes as measured by flow cytometry as described in Materials and Methods. One representative histogram is shown for each cell surface marker. (TIFF) [file pntd.0012417.s003.tiff]
