# Supplementary material for: Altered co-stimulatory and inhibitory receptors on monocyte subsets in patients with visceral leishmaniasis
Source: PLoS Negl Trop Dis. 2024 Aug 19;18(8):e0012417. doi: 10.1371/journal.pntd.0012417 (PMC11373857; doi:10.1371/journal.pntd.0012417)
Supplement: S1 Table — PBMCs were purified from VL patients at ToD (n = 20) and EoT (n = 20) and from HNEC (n = 10) and the expression levels (MFI = Median Fluorescence Intensity) of CD40 were measured on the different monocyte subsets by flow cytometry. Results are presented as median with interquartile range. Statistical differences were determined by Kruskall-Wallis test (*) and Dunn’s multiple comparisons test (#). ToD = Time of Diagnosis; EoT = End of Treatment; HNEC = healthy non-endemic controls. C = classical monocytes. I = intermediate monocytes. NC = non-classical monocytes. (DOCX) [file pntd.0012417.s004.docx]

**S1 Table: CD40 MFI on monocyte subsets from VL patients at ToD and EoT and on monocytes from HNEC**

| **ToD** | **CD40 MFI**  **(x10^3^)** | ***p value** | **Comparisons**  **CD40 MFI** | **^#^p value** |
| --- | --- | --- | --- | --- |
| Classical | 13 [11-14.9] | <0.0001 | C vs I | 0.0007 |
| Intermediate | 22.2 [16.9-26.7] |  | C vs NC | 0.4656 |
| Non-classical | 5.2 [1.2-14] |  | I vs NC | <0.0001 |
| **EoT** | **CD40 MFI**  **(x10^3^)** | ***p value** | **Comparisons**  **CD40 MFI** | **^#^p value** |
| Classical | 5 [4-6.3] | 0.0001 | C vs I | 0.0034 |
| Intermediate | 9.6 [6.6-11] |  | C vs NC | >0.9999 |
| Non-classical | 2.0 [1.5-8 |  | I vs NC | 0.0002 |
| **HNEC** | **CD40 MFI**  **(x10^3^)** | ***p value** | **Comparisons**  **CD40 MFI** | **^#^p value** |
| Classical | 2.1 [1.7-3.5] | 0.1050 | C vs I | 0.1265 |
| Intermediate | 5 [2.5-6.4] |  | C vs NC | 0.3639 |
| Non-classical | 3.4 [2.7-4.3] |  | I vs NC | >0.9999 |
