# Supplementary material for: Altered co-stimulatory and inhibitory receptors on monocyte subsets in patients with visceral leishmaniasis
Source: PLoS Negl Trop Dis. 2024 Aug 19;18(8):e0012417. doi: 10.1371/journal.pntd.0012417 (PMC11373857; doi:10.1371/journal.pntd.0012417)
Supplement: S2 Table — PBMCs were purified from VL patients at ToD (n = 17) and EoT (n = 17) and from HNEC (n = 10) and the expression levels (MFI = Median Fluorescence Intensity) of CD80 were measured on the different monocyte subsets by flow cytometry. Results are presented as median with interquartile range. Statistical differences were determined by Kruskall-Wallis test (*) and Dunn’s multiple comparisons test (#). ToD = Time of Diagnosis; EoT = End of Treatment; HNEC = healthy non-endemic controls. C = classical monocytes. I = intermediate monocytes. NC = non-classical monocytes. (DOCX) [file pntd.0012417.s005.docx]

**Table S2: CD80 MFI on monocyte subsets from VL patients at ToD and EoT and on monocytes from HNEC**

| **ToD** | **CD80 MFI** | ***p value** | **Comparisons**  **CD80 MFI** | **^#^p value** |
| --- | --- | --- | --- | --- |
| Classical | 1321 [998-1579] | 0.0021 | C vs I | 0.0020 |
| Intermediate | 2060 [1506-3062] |  | C vs NC | 0.0434 |
| Non-classical | 1826 [1356-2289] |  | I vs NC | >0.9999 |
| **EoT** | **CD80 MFI** | ***p value** | **Comparisons**  **CD80 MFI** | **^#^p value** |
| Classical | 1039 [930-1260] | <0.0001 | C vs I | 0.0081 |
| Intermediate | 1445 [1231-1791] |  | C vs NC | <0.0001 |
| Non-classical | 1630 [1464-1993] |  | I vs NC | 0.3922 |
| **HNEC** | **CD80 MFI** | ***p value** | **Comparisons**  **CD80 MFI** | **^#^p value** |
| Classical | 807 [657-934] | 0.0042 | C vs I | 0.0925 |
| Intermediate | 1120 [937-1420] |  | C vs NC | 0.0034 |
| Non-classical | 1325 [1183-1706] |  | I vs NC | 0.8242 |
