# Supplementary material for: Altered co-stimulatory and inhibitory receptors on monocyte subsets in patients with visceral leishmaniasis
Source: PLoS Negl Trop Dis. 2024 Aug 19;18(8):e0012417. doi: 10.1371/journal.pntd.0012417 (PMC11373857; doi:10.1371/journal.pntd.0012417)
Supplement: S3 Table — PBMCs were purified from VL patients at ToD (n = 20) and EoT (n = 20) and from HNEC (n = 10) and the expression levels (MFI = Median Fluorescence Intensity) of CD86 were measured on the different monocyte subsets by flow cytometry. Results are presented as median with interquartile range. Statistical differences were determined by Kruskall-Wallis test (*) and Dunn’s multiple comparisons test (#). ToD = Time of Diagnosis; EoT = End of Treatment; HNEC = healthy non-endemic controls. C = classical monocytes. I = intermediate monocytes. NC = non-classical monocytes. (DOCX) [file pntd.0012417.s006.docx]

**Table S3: CD86MFI on monocyte subsets from VL patients at ToD and EoT and on monocytes from HNEC**

| **ToD** | **CD86 MFI** | ***p value** | **Comparisons**  **CD86 MFI** | **^#^p value** |
| --- | --- | --- | --- | --- |
| Classical | 5606 [4646-7007] | 0.0003 | C vs I | 0.0008 |
| Intermediate | 9500 [7688-12461] |  | C vs NC | >0.9999 |
| Non-classical | 3604 [1054-10332] |  | I vs NC | 0.0036 |
| **EoT** | **CD86 MFI** | ***p value** | **Comparisons**  **CD86 MFI** | **^#^p value** |
| Classical | 6051 [4133-7919] | <0.0001 | C vs I | <0.0001 |
| Intermediate | 11569 [8234-13889] |  | C vs NC | <0.0001 |
| Non-classical | 11877 [8136-13303] |  | I vs NC | >0.9999 |
| **HNEC** | **CD86 MFI** | ***p value** | **Comparisons**  **CD86 MFI** | **^#^p value** |
| Classical | 4374 [3587-5841] | 0.0035 | C vs I | 0.0063 |
| Intermediate | 8436 [6258-9775] |  | C vs NC | 0.0197 |
| Non-classical | 7600 [6065-9769] |  | I vs NC | >0.9999 |
