# Supplementary material for: Altered co-stimulatory and inhibitory receptors on monocyte subsets in patients with visceral leishmaniasis
Source: PLoS Negl Trop Dis. 2024 Aug 19;18(8):e0012417. doi: 10.1371/journal.pntd.0012417 (PMC11373857; doi:10.1371/journal.pntd.0012417)
Supplement: S4 Table — PBMCs were purified from VL patients at ToD (n = 20) and EoT (n = 20) and from HNEC (n = 10) and the expression levels (MFI = Median Fluorescence Intensity) of PD-L1 were measured on the different monocyte subsets by flow cytometry. Results are presented as median with interquartile range. Statistical differences were determined by Kruskall-Wallis test (*) and Dunn’s multiple comparisons test (#). ToD = Time of Diagnosis; EoT = End of Treatment; HNEC = healthy non-endemic controls. C = classical monocytes. I = intermediate monocytes. NC = non-classical monocytes. (DOCX) [file pntd.0012417.s007.docx]

**Table S4: PD-L1 MFI on monocyte subsets from VL patients at ToD and EoT and on monocytes from HNEC**

| **ToD** | **PD-L1 MFI** | ***p value** | **Comparisons**  **PD-L1 MFI** | **^#^p value** |
| --- | --- | --- | --- | --- |
| Classical | 2951 [2132-4051] | 0.0001 | C vs I | 0.0008 |
| Intermediate | 5779 [4578-8411] |  | C vs NC | >0.9999 |
| Non-classical | 3451 [1183-4191] |  | I vs NC | 0.0007 |
| **EoT** | **PD-L1 MFI** | ***p value** | **Comparisons**  **PD-L1 MFI** | **^#^p value** |
| Classical | 1233 [1009-2076] | 0.0131 | C vs I | 0.0099 |
| Intermediate | 2058 [1656-3169] |  | C vs NC | 0.3038 |
| Non-classical | 1923 [1365-2506] |  | I vs NC | 0.5816 |
| **HNEC** | **PD-L1 MFI** | ***p value** | **Comparisons**  **PD-L1 MFI** | **^#^p value** |
| Classical | 893 [687-1205] | 0.0572 | C vs I | 0.0668 |
| Intermediate | 1269 [996-2233] |  | C vs NC | 0.2390 |
| Non-classical | 1212 [887-1604] |  | I vs NC | >0.9999 |
